# Inflammasomes: a rising star on the horizon of COVID-19 pathophysiology

**DOI:** 10.3389/fimmu.2023.1185233

**Published:** 2023-05-12

**Authors:** Man Wang, Fei Yu, Wenguang Chang, Yuan Zhang, Lei Zhang, Peifeng Li

**Affiliations:** Institute for Translational Medicine, The Affiliated Hospital of Qingdao University, College of Medicine, Qingdao University, Qingdao, China

**Keywords:** SARS-CoV-2, COVID-19, inflammasomes, proinflammatory cytokines, immunopathogenesis, inflammasome-targeted therapies

## Abstract

Severe acute respiratory syndrome coronavirus 2 (SARS-CoV-2) is a contagious respiratory virus that is the cause of the coronavirus disease 2019 (COVID-19) pandemic which has posed a serious threat to public health. COVID-19 is characterized by a wide spectrum of clinical manifestations, ranging from asymptomatic infection to mild cold-like symptoms, severe pneumonia or even death. Inflammasomes are supramolecular signaling platforms that assemble in response to danger or microbial signals. Upon activation, inflammasomes mediate innate immune defense by favoring the release of proinflammatory cytokines and triggering pyroptotic cell death. Nevertheless, abnormalities in inflammasome functioning can result in a variety of human diseases such as autoimmune disorders and cancer. A growing body of evidence has showed that SARS-CoV-2 infection can induce inflammasome assembly. Dysregulated inflammasome activation and consequent cytokine burst have been associated with COVID-19 severity, alluding to the implication of inflammasomes in COVID-19 pathophysiology. Accordingly, an improved understanding of inflammasome-mediated inflammatory cascades in COVID-19 is essential to uncover the immunological mechanisms of COVID-19 pathology and identify effective therapeutic approaches for this devastating disease. In this review, we summarize the most recent findings on the interplay between SARS-CoV-2 and inflammasomes and the contribution of activated inflammasomes to COVID-19 progression. We dissect the mechanisms involving the inflammasome machinery in COVID-19 immunopathogenesis. In addition, we provide an overview of inflammasome-targeted therapies or antagonists that have potential clinical utility in COVID-19 treatment.

## 1 Introduction

**Figure 1:**
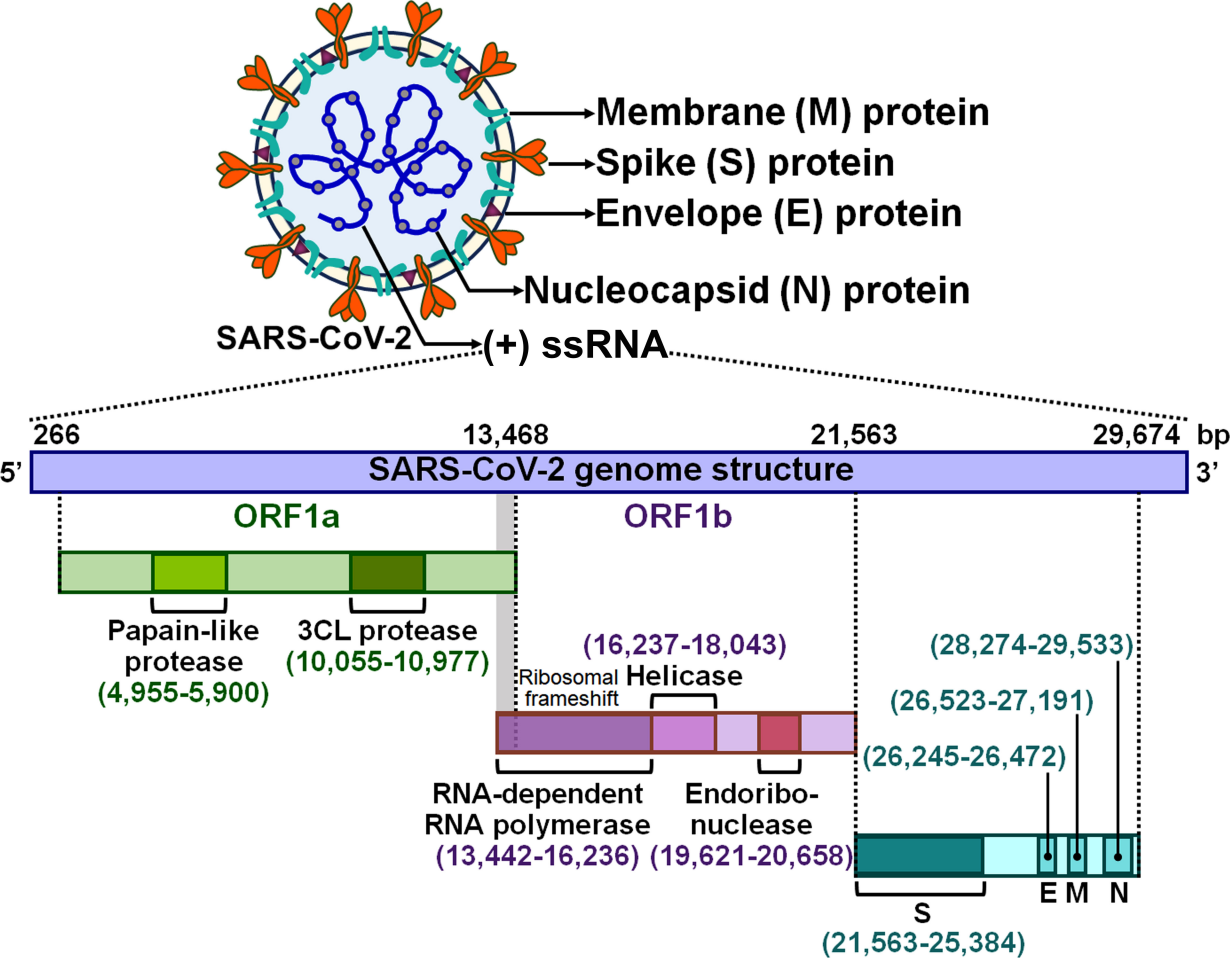
Schematic representation of the SARS-CoV-2 virion and genomic structure. SARS-CoV-2 is an enveloped +ssRNA virus with a genome of 29,674 bp. The SARS-CoV-2 virion has four main structural proteins, which are spike (S), envelope (E), membrane (M) and nucleocapsid (N) glycoproteins. The viral genome contains several ORFs that encode nonstructural proteins and structural proteins. Two large overlapping ORFs (ORF1a and ORF1b) occupy two-thirds of the viral genome at the 5’-end. ORF1a encodes papain-like protease and 3CL protease, while ORF1b encodes RNA-dependent RNA polymerase, helicase and endoribonuclease. A third of the genome at the 3’-end encodes the four essential structural proteins (S, E, M and N). +ssRNA, single-stranded positive-sense RNA; ORF, open reading frame; 3CL protease, 3-chymotrypsin-like protease; S, spike; E, envelope; M, membrane; N, nucleocapsid.

Severe acute respiratory syndrome coronavirus 2 (SARS-CoV-2) is the causative agent responsible for the ongoing coronavirus disease 2019 (COVID-19) pandemic (1). SARS-CoV-2 is an enveloped single-stranded positive-sense RNA (+ssRNA) virus with a genome of approximately 30 kb (2) (**Figure 1**). Like other coronaviruses, SARS-CoV-2 virions are comprised of four major structural proteins, spike (S), membrane (M), nucleocapsid (N) and envelope (E) proteins (3). The viral genome contains various open reading frames (ORFs) encoding for nonstructural proteins (NSPs, e.g., 3-chymotrypsin-like cysteine (3CL) protease and RNA-dependent RNA polymerase) and the four essential structural proteins (4). SARS-CoV-2 is principally transmitted via respiratory droplets, aerosols, and to a lesser extent, direct contact with fomites from infected people (5). Albeit the respiratory system is the primary target of SARS-CoV-2, this virus can also damage other vital organs including brain, gut, heart and kidney (6, 7). Typical manifestations of COVID-19 encompass cough, fatigue, fever, headache and shortness of breath (8). Most SARS-CoV-2-infected individuals have mild symptoms such as cough and fever, while some experience severe pneumonia and acute respiratory distress syndrome (ARDS) with systemic inflammation (9). The systemic inflammatory syndrome is triggered by over-activation of innate immune system and intense proinflammatory cytokine responses (10). Generally, mild cases of COVID-19 can be cured using over-the-counter medications (e.g., paracetamol), but patients with severe COVID-19 have a high risk of life-threatening complications and death due to a lack of specific therapeutic options (11). The nature of host immune response to SARS-CoV-2 could have a tremendous impact on COVID-19 progression. Elucidating the immunological mechanisms responsible for disease severity should be a focus of SARS-CoV-2 research with the aim of improving clinical outcomes in COVID-19 patients.

Established studies have proven that SARS-CoV-2 represents a pathogen-associated molecular pattern (PAMP) able to trigger inflammasome activation (12, 13). Inflammasomes are macromolecular immune complexes formed in the cytosol in response to an array of stimuli (14). The assembly of inflammasomes is initiated by pattern-recognition receptors (PRRs) following the detection of endogenous or pathogenic danger signals (15). Activated inflammasomes fuel the inflammatory caspase to induce pyroptosis and cytokine production, thereby removing virus-infected cells from the host (16–18). However, aberrant inflammasome activation can drive metainflammation and bring about deleterious consequences. Currently, considerable attention has been paid to the involvement of inflammasomes in the pathogenesis of various diseases such as cancer, cardiovascular disorders and diabetes (19–21). Aberrant inflammasome activation and concomitant cytokine burst also occur in COVID-19 patients (22, 23). More importantly, COVID-19 progression and severity have been closely correlated with the active status of inflammasomes and the levels of proinflammatory cytokines including interleukin (IL)-1β and IL-18 (24, 25), stressing the potential role of inflammasomes in the pathophysiology of COVID-19-associated systemic complications. Recent studies have contributed towards an advanced understanding of the interaction between SARS-CoV-2 and inflammasomes throughout the course of infection. Here, we review the literature concerning the mechanisms behind SARS-CoV-2-mediated regulation of inflammasome activation, the role of inflammasomes in COVID-19 immunopathogenesis and the potential mechanisms that coordinate inflammasome-directed pathologies in COVID-19. In addition, we discuss the therapeutic potential of inflammasome-centered therapies. Future research into inflammasome activity will provide new perspectives for illuminating the pathological processes underlying COVID-19 progression and facilitate the development of more targeted therapeutics for this infectious disease.

## 2 Inflammasomes

**Figure 2:**
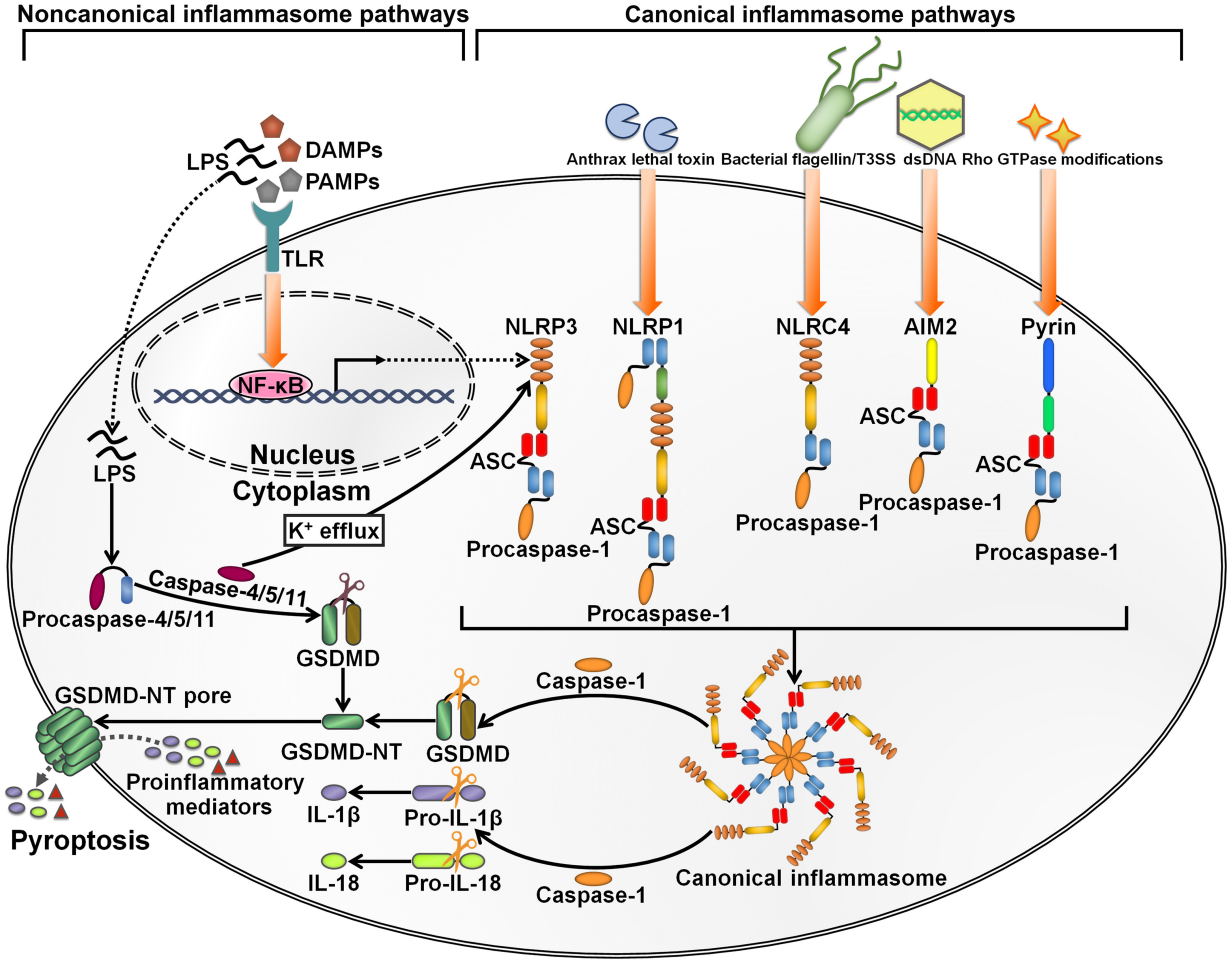
Schematic illustration of key inflammasome pathways. Canonical inflammasomes are multimeric protein complexes in the cytosol that can be activated by diverse signals. They are generally composed of a cytosolic sensor (e.g., PRR), an adaptor protein (ASC) and an effector protein (e.g., procaspase-1). NLRP3 inflammasome activation is caused by a number of DAMPs and PAMPs, such as ATP, nucleic acids, bacteria, fungi and viruses. NLRP1 is mainly triggered by anthrax lethal toxin and muramyl dipeptide. NLRC4 is activated by bacterial flagellin and type III secretion apparatus. AIM2 typically responds to cytosolic dsDNA. Pyrin detects inactivating modifications of host Rho GTPases by diverse bacterial effectors or toxins. The assembly of canonical inflammasomes leads to caspase-1 activation. Caspase-1 then cleaves GSDMD to unleash its N-terminal pore-forming domain (GSDMD-NT). GSDMD-NT punches holes in the cellular membrane, which induces water influx, cell swelling and cytomembrane rupture, ultimately leading to the occurrence of an inflammatory cell death termed pyroptosis. Caspase-1 also mediates the conversion of pro-IL-1β and pro-IL-18 into their active forms. The bioactive IL-1β and IL-18 are released into the extracellular space via the GDDMD-NT pores. Cell lysis further enhances the inflammatory signaling through extravasation of DAMPs. The noncanonical inflammasome pathway is initiated by LPS from extracellular Gram-negative bacteria. LPS directly binds to procaspase-4/5 (human) or procaspase-11 (mouse) to induce their activation. Active caspase-4/5/11 cleave GSDMD into GSDMD-NT, hence actuating the pyroptosis program. Furthermore, caspase-4/5/11 can motivate the NLRP3 inflammasome pathway for inflammatory cytokine processing by promoting K^+^ efflux. LPS, lipopolysaccharide; DAMPs, damage-associated molecular patterns; PAMPs, pathogen-associated molecular patterns; TLR, Toll-like receptor; NF-κB, nuclear factor-κB; GSDMD, gasdermin D; GSDMD-NT, the N-terminal fragment of gasdermin D; IL-1β, interleukin-1β; IL-18, interleukin-18; NLRP3, nucleotide-binding oligomerization domain-like receptor family pyrin domain-containing protein 3; ASC, apoptosis-associated speck-like protein containing a caspase recruitment domain; NLRP1, nucleotide-binding oligomerization domain-like receptor family pyrin domain-containing protein 1; NLRC4, nucleotide-binding oligomerization domain-like receptor family caspase recruitment domain-containing protein 4; dsDNA, double-stranded DNA; AIM2, absent in melanoma 2.

Inflammasomes are intracellular multimeric innate immune rheostats that trigger inflammation in response to multiple physiological or pathogenic factors (26). Inflammasomes mediate the proteolytic maturation of proinflammatory cytokines IL-1β and IL-18, culminating in an expeditious inflammatory form of programmed cell death termed pyroptosis (27). The inflammasomes can be categorized into canonical and noncanonical inflammasomes (28) (**Figure 2**). Canonical inflammasomes encompassing absent in melanoma 2 (AIM2), nucleotide-binding oligomerization domain (NOD)-like receptor family pyrin domain (PYD)-containing protein 1 (NLRP1), NLRP3, NOD-like receptor (NLR) family caspase recruitment domain (CARD)-containing protein 4 (NLRC4) and pyrin can motivate caspase-1 (29). Noncanonical inflammasomes serve as platforms for the activation of caspase-4/5 (human) or caspase-11 (mouse) (30). Canonical inflammasomes typically consist of three major components: an upstream cytosolic sensor (e.g., PRR), an adaptor protein (e.g., apoptosis-associated speck-like protein containing a caspase recruitment domain (ASC)) and a downstream effector protein (e.g., procaspase-1) (31). The structures of canonical inflammasomes are similar, and the main discrepancy lies in the diversity of cytosolic sensors (32). Cytosolic PRRs expressed by innate immune cells include C-type lectin receptors (CLRs), NLRs and Toll-like receptors (TLRs) that can sense damage-associated molecular patterns (DAMPs) or PAMPs to prime inflammasome assembly (33). The sensor proteins NLRP1, NLRP3 and NLRC4, which form canonical inflammasomes, are members of the NLR family, while AIM2 belongs to the ALR family (34). The sensor proteins react to a multitude of signals. NLRP1 is able to detect anthrax lethal toxin and muramyl dipeptide (35, 36). NLRP3, one of the most studied sensor proteins, responds to a number of agonists including adenosine triphosphate (ATP), nucleic acids, bacteria, fungi and viruses (37). NLRC4 senses bacterial flagellin and type III secretion apparatus in the cytosol (38, 39). AIM2 is activated by cytosolic double-stranded DNA (dsDNA) (40), while pyrin recognizes inactivating modifications of host Rho GTPases by diverse bacterial effectors or toxins, including adenylylation, deamidation and glucosylation (41). CARD-carrying PRRs (e.g., NLRP1 and NLRC4) directly recruit procaspase-1 to initiate inflammasome assembly. In ASC-independent inflammasomes, caspase-1 is activated via dimerization-induced autoproteolysis (42). ASC, which contains an N-terminal PYD and a C-terminal CARD, serves as a scaffold protein bridging PYD-containing PRRs (e.g., AIM2, NLRP3 and pyrin) and procaspase-1 (43). Upon activation, PYD-carrying PRRs couple to ASC via homotypic interactions and drive the supramolecular assembly of ASC, leading to the formation of ASC specks (44). ASC specks subsequently recruit inactive zymogen procaspase-1 via CARD-CARD interactions, which brings monomers of procaspase-1 in close proximity and facilitates its autoproteolysis (45). Active caspase-1 not only proteolytically processes the biologically inactive procytokines (pro-IL-1β and pro-IL-18) into the active mature forms, but also cleaves gasdermin D (GSDMD) to unmask its N-terminal pore-forming domain (GSDMD-NT) (46, 47). GSDMD-NT perforates the cellular membrane and induces pyroptosis, leading to the secretion of proinflammatory cytokines into the extracellular milieu (48). The canonical inflammasome-dependent pyroptosis constitutes a significant innate defense mechanism against pathogen invasion. In the noncanonical inflammasome pathway, procaspase-4/5/11 can directly bind to bacterial lipopolysaccharide (LPS) to activate their proteolytic activity (49). Caspase-4/5/11 cleave GSDMD to induce pyroptosis (46, 50). Caspase-11 is capable of stimulating the NLRP3 inflammasome by fostering potassium (K^+^) efflux (51). After that, active caspase-1 promotes the maturation of proinflammatory cytokines, which can be released to the outside of the cell through GSDMD-NT pores.

## 3 Inflammasome activation during SARS-CoV-2 infection

Inflammasomes play a double-edged role in viral infection. Appropriate activation of inflammasomes instigates innate antiviral responses, but the dysregulation of the inflammasome signaling due to defective host immunity or comorbidities can lead to the cytokine storm and hyperinflammation. NLRP3 has been detected in various cell types, including innate immune cells, lung epithelial cells and cardiac cells (52). It is thus inferred that SARS-CoV-2 infection could cause NLRP3 inflammasome activation in these cells.

### 3.1 Peripheral blood mononuclear cells

Reportedly, NLRP3 inflammasome was activated in peripheral blood mononuclear cells (PBMCs) from COVID-19 patients (12). The levels of active caspase-1 and IL-18 were higher in the sera of COVID-19 patients than the controls (**Table 1**). The serum concentration of active caspase-1 showed a positive association with the levels of C-reactive protein (CRP), IL-6, IL-18 and lactate dehydrogenase (LDH). Moreover, NLRP3 inflammasome activation had an impact on the clinical outcome of COVID-19. Particularly, the level of active caspase-1 was elevated as illness severity increased. The level of IL-18 was higher in patients who required mechanical ventilation than patients who did not. Likewise, lethal cases had an increased level of IL-18 relative to survivors. Since caspase-1-independent pathway also gives rise to enhanced IL-18 secretion (61), the relationship between NLRP3 inflammasome activation and COVID-19 development is an important subject to be further addressed. The robust activation of NLRP3 inflammasome may aggrandize inflammation in patients with severe COVID-19, culminating in worse clinical results. The NLRP3 inflammasome could be used as a prospective predictor of COVID-19 severity and outcomes. Blockade of inflammasome pathways represents a promising approach to treat patients with severe COVID-19. An improved understanding of activated inflammasome complexes during the course of COVID-19 will offer new opportunities for therapeutic intervention.

**Table 1:** Overview of altered cytokine profiles in COVID-19 patients.

Author	Year	GSCF	IFN-γ	IL-1β	IL-1RA	IL-2	IL-4	IL-6	IL-7	IL-8	IL-10	IL-18	CXCL10	MCP-1	MIP-1α	TNF-α	Reference
Rodrigues et al.	2021		↓	↑			↑	↑			↑	↑					(12)
Courjon et al.	2021			↑	↑							↑					(53)
Leal et al.	2023							↑		↑		↑					(54)
Bao et al.	2020			↑				↑			↑						(55)
Huang et al.	2020	↑	↑	↑	↑	↑			↑	↑	↑		↑	↑	↑	↑	(56)
Ulhaq et al.	2020							↑									(57)
Ren et al.	2021		↓	↑				↑								↑	(58)
Zhan et al.	2021							↑			↑						(59)
Carstens et al.	2022			↑								↑				↑	(60)

↑represents increased expression level in COVID-19 patients versus healthy subjects; ↓represents decreased expression level in COVID-19 patients versus healthy subjects; GSCF, granulocyte colony stimulating factor; IFN-γ, interferon-γ; IL-1β, interleukin-1β; IL-1RA, interleukin-1 receptor antagonist; IL-2, interleukin-2; IL-4, interleukin-4; IL-6, interleukin-6; IL-7, interleukin-7; IL-8, interleukin-8; IL-10, interleukin-10; IL-18, interleukin-18; CXCL10, C-X-C motif chemokine ligand 10; MCP-1, monocyte chemoattractant protein-1; MIP-1α, macrophage inflammatory protein-1α; TNF-α, tumor necrosis factor-α.

### 3.2 Myeloid cells

Myeloid cells are a vital cellular compartment of innate immunity and consist of monocytes, macrophages and neutrophils (62). Myeloid cells isolated from COVID-19 patients showed heterogeneous NLRP3 inflammasome activation potentials (53). Opposed potentials of caspase-1 activation were observed between nonclassical monocytes and immature neutrophils from severe/critical COVID-19 patients upon NLRP3 inflammasome stimulation. NLRP3 inflammasome activation of nonclassical monocytes was elevated with increasing COVID-19 severity, while CD66b^+^CD16^dim^ granulocytes exhibited lower NLRP3 activation potential in severe forms of COVID-19 than mild cases. The number of nonclassical monocytes was remarkably decreased in severe to critical patients compared with healthy controls, while immature neutrophils exhibited the opposite trend. In recovered patients, caspase-1 activation in CD66b^+^CD16^dim^ granulocytes was restored and the percentage of immature neutrophils was comparable to the controls. Altogether, the NLRP3 inflammasome signatures in myeloid cells may represent a predictive biomarker for COVID-19 development and patient outcome and provide important guidance for therapeutic decisions involving immunoregulatory agents.

Monocytes and macrophages act as sentinel cells that detect SARS-CoV-2 infection to assemble inflammasomes (e.g., AIM2 and NLRP3), resulting in pyroptosis and the extravasation of proinflammatory mediators (63). CD14^high^CD16^-^ monocytes derived from severe COVID-19 patients showed increased NLRP3 inflammasome activation potential, as shown by the formation of ASC speck/caspase-1 complexes and increased IL-1β secretion (64). Monocytes from critically ill patients with COVID-19 exhibited higher caspase-1 activation than those from healthy controls (13). Accordingly, intense monocyte death was documented in critically ill patients. The plasma concentration of IL-1β was higher in critically ill patients than healthy controls. Inflammasome activation in monocytes caused massive production of proinflammatory cytokines, leading to hyperinflammation in severe COVID-19. These findings implied that COVID-19 deterioration was associated with inflammasome activation in monocytes. Monocytes may be pivotal cells in the detrimental proinflammatory cascades that reflect COVID-19 progression and severity. SARS-CoV-2 infection instigated the NLRP3 inflammasome pathway in human lung macrophages (24). MCC950, a selective inhibitor of NLRP3, could dampen the release of proinflammatory cytokines and chemokines; in parallel, it reduced inflammation and reversed chronic lung pathology in SARS-CoV-2-infected mice. In addition, SARS-CoV-2 open reading frame 3a (ORF3a) protein was able to activate the NLRP3 inflammasome in LPS-primed macrophages, and it could promote the expression of pro-IL-1β via nuclear factor-κB (NF-κB) (65). A recent study indicated that NLRP3 inflammasome was hyperactivated in neutrophils from severe COVID-19 patients (54). Severe COVID-19 neutrophils exhibited impaired responsiveness to NLRP3 insults (e.g., ATP and bacterial LPS). These NLRP stimuli failed to induce the expression of NLRP3 inflammasome components, which might involve an epigenetic mechanism. The presence of numerous immature neutrophils may be an explanation for COVID-19 neutrophil dysfunction. The reduced neutrophilic responsiveness towards common NLRP3 stimuli could predispose severe COVID-19 patients to secondary pathogen infections. The mechanism responsible for alterations in neutrophilic response to NLRP3 stimulation necessitates considerable attention. It will be of paramount importance to clarify the causal link between neutrophil impairment and COVID-19 immunopathogenesis.

### 3.3 Endothelial cells

Notably, circulatory exosomes from severe COVID-19 patients were able to increase the expression of NLRP3, caspase-1 and IL-1β in endothelial cells (66). COVID-19 exosomes markedly promoted IL-1β secretion. These results demonstrated that exosomes from COVID-19 plasma stimulated NLRP3 inflammasome in distant endothelial cells, leading to amplified inflammatory reactions. Endothelial cells function as a selectively permeable barrier to blood and dominate the transfer of proteins from the circulation to tissues (67). Endothelial cells act to oppose inflammation. Dysfunction of endothelial cells can give rise to multiorgan damage that is a general characteristic of viral infections (68). Exosomes act as a crucial mediator for endothelial cell dysfunction and multiorgan inflammation during SARS-CoV-2 infection. As exosomes carry a number of bioactive materials, it is intriguing which components have the ability to ignite NLRP3 inflammasome. The mechanism responsible for COVID-19 exosome-induced NLRP3 inflammasome activation is worthy of more detailed investigation.

## 4 The regulatory mechanisms of SARS-CoV-2 in inflammasome activation

**Figure 3:**
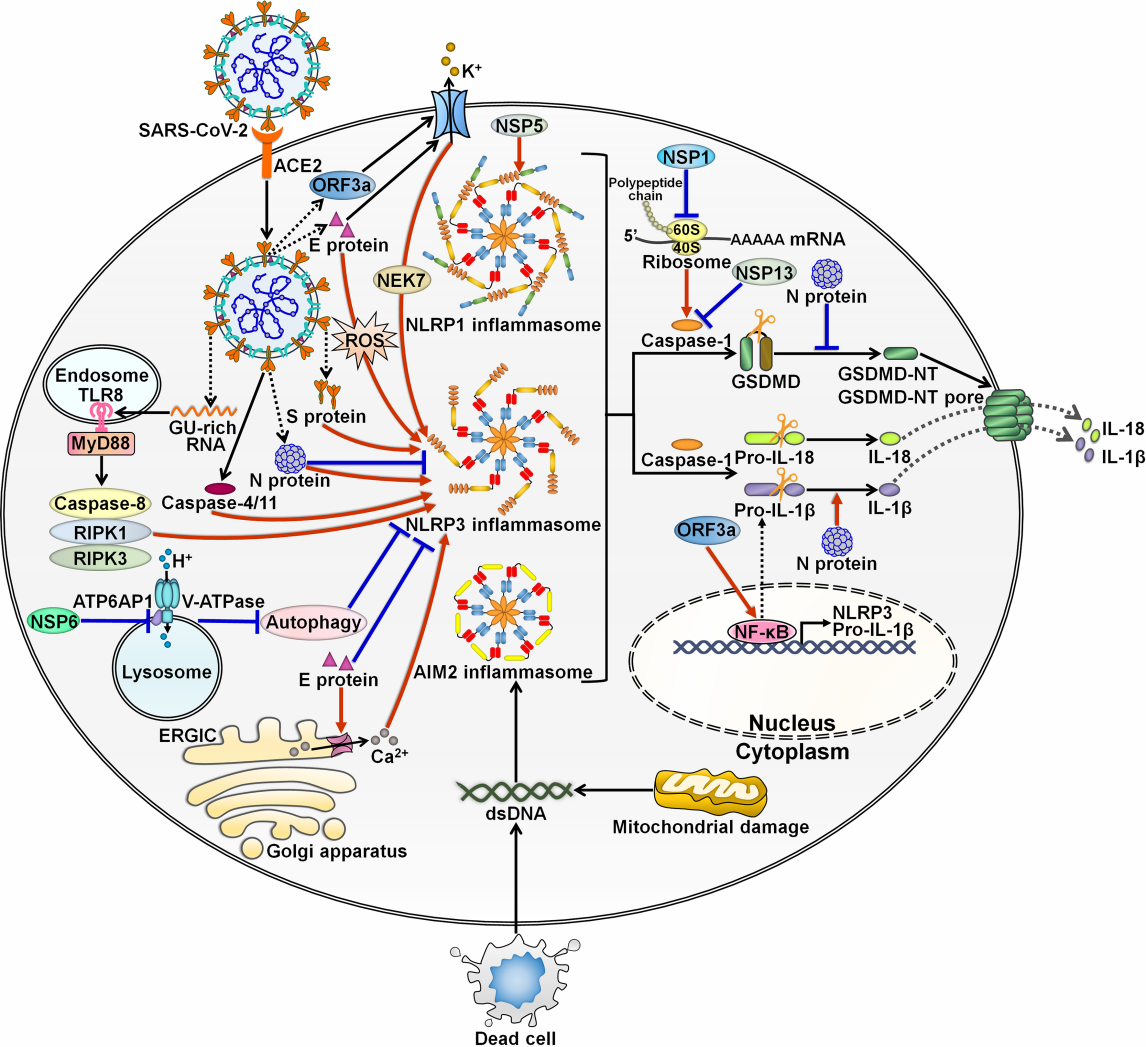
Regulation of inflammasome pathways by SARS-CoV-2. SARS-CoV-2 infection triggers the assembly of inflammasomes. These molecular platforms impel caspase-1 activation, hence promoting the extracellular secretion of IL-1β and IL-18. SARS-CoV-2 has evolved a multitude of mechanisms to interfere with inflammasome activity. Viral ORF3a and E proteins can activate NLRP3 inflammasome by inducing K^+^ efflux. ORF3a increases the expression of pro-IL-1β by motivating the NF-κB signaling cascade. E protein triggers the formation of Ca^2+^ ion channels in ERGIC/Golgi membranes and initiates ROS-dependent activation of NLRP3 inflammasome. Paradoxically, E protein is found to exert inhibitory effects on NLRP3 inflammasome priming by blunting ER stress. S protein acts as an agonist of NLRP3 inflammasome. The GU-rich RNA derived from S protein actuates NLRP3 inflammasome through the caspase-8/RIPK1/RIPK3 signaling pathway. SARS-CoV-2 infection can induce NLRP3 inflammasome formation by upregulating caspase-4/11. N protein plays dual roles in regulating NLRP3 inflammation. N protein favors the assembly of NLRP3 inflammasome and drives the maturation of the proinflammatory cytokine IL-1β. N protein blocks the NLRP3 inflammasome signaling by preventing caspase-1-mediated GSDMD activation. NSP6 suppresses lysosome acidification through direct interaction with ATP6AP1, causing autophagic flux stagnation to activate NLRP3 inflammasome. NSP1 and NSP13 repress NLRP3 inflammasome activation and IL-1β maturation via attenuation of caspase-1 activity. Specifically, NSP1 impedes host translation by binding to the 40S ribosome subunit, which is required for caspase-1 inhibition. Notably, viral NSP5 can induce NLRP1 inflammasome activation. SARS-CoV-2 infection also contributes to the assembly of AIM2 inflammasome. SARS-CoV-2-induced excessive cell death results in the liberation of endogenous DNA. Moreover, SARS-CoV-2 may favor the release of mtDNA into the cytoplasm by impairing mitochondrial membranes. Either free DNA or mtDNA can activate AIM2 inflammasome. SARS-CoV-2, severe acute respiratory syndrome coronavirus 2; ACE2, angiotensin-converting enzyme 2; ORF3a, open reading frame 3a; E protein, envelope protein; TLR8, Toll-like receptor 8; MyD88, myeloid differentiation factor 88; RIPK1, receptor-interacting protein kinase 1; RIPK3, receptor-interacting protein kinase 3; NSP6, nonstructural protein 6; ATP6AP1, ATPase H^+^ transporting accessory protein 1; ERGIC, endoplasmic reticulum Golgi apparatus intermediate compartment; ROS, reactive oxygen species; S protein, spike protein; N protein, nucleocapsid protein; NSP5, nonstructural protein 5; NEK7, NIMA-related kinase 7; NLRP1, nucleotide-binding oligomerization domain-like receptor family pyrin domain-containing protein 1; NLRP3, nucleotide-binding oligomerization domain-like receptor family pyrin domain-containing protein 3; AIM2, absent in melanoma 2; dsDNA, double-stranded DNA; NSP1, nonstructural protein 1; NSP13, nonstructural protein 13; GSDMD, gasdermin D; GSDMD-NT, the N-terminal fragment of gasdermin D; IL-1β, interleukin-1β; IL-18, interleukin-18; NF-κB, nuclear factor-κB.

Accumulating evidence has indicated the complex interaction between SARS-CoV-2 and inflammasomes. At the initial stage of infection, SARS-CoV-2 and its derived products stimulate inflammasome formation, inducing inflammatory responses to fight against viral infection. SARS-CoV-2 employs diverse strategies to hinder inflammasome activation, ensuring a beneficial environment for its replication. After that, SARS-CoV-2 inflames the inflammatory reaction to propel viral pathogenicity. Highly sophisticated mechanisms involving various viral components contribute to regulation of inflammasome functionality during SARS-CoV-2 infection (**Figure 3**).

### 4.1 Mechanisms of SARS-CoV-2-mediated inhibition of inflammasome activation

The inflammasome signaling performs important functions in pathogen defense. It is conceived that SARS-CoV-2 has evolved multiple mechanisms to counteract inflammasome-dependent cascades, thereby allowing for viral survival and pathogenesis. SARS-CoV-2 N protein was released into the cytosol immediately after viral entry into the host cell (69). N protein inhibited pyroptotic cell death in infected human monocytes by binding to GSDMD linker region and impeding caspase-1-mediated GSDMD activation. This event prevented the release of immune signaling factors into the extracellular milieu. Because of GSDMD inhibition, excessive proinflammatory cytokines might accumulate in the host cytosol. The release of massive proinflammatory cytokines could result in severe disease in COVID-19 patients due to the lytic infection of SARS-CoV-2. The interfaces between viral N protein and GSDMD were diverse, and both the N-terminal and C-terminal domains of GSDMD were required for the suppression of GSDMD activation, suggesting a higher tertiary structure employed by N protein to seize GSDMD. The impact of SARS-CoV-2 N protein on the inflammatory response and COVID-19 progression should be further explored. SARS-CoV-2 E protein had the ability to inhibit endoplasmic reticulum (ER) stress and NLRP3 inflammasome priming in macrophages (70). In mice treated with poly(I:C) to simulate the effects of viral infection, E protein decreased the expression levels of IL-1β and IL-18 in bronchoalveolar lavage fluid (BALF) and suppressed macrophage infiltration in the lung tissues. Thus, E protein prevented NLRP3 inflammasome activation and pulmonary inflammation. Two early products of SARS-CoV-2 infection, NSP1 and NSP13, were revealed to suppress NLRP3 inflammasome activation and IL-1β maturation via reduction of caspase-1 activity (71). Mechanistic investigation indicated that NSP1 induced RNA cleavage and suppressed host translation by binding to the 40S ribosome subunit, which was required for caspase-1 inhibition. NSP1 potentially prevented NLRP3 inflammasome assembly by downregulating its constituents, including NLRP3, ASC and caspase-1. As for NSP13, its N-terminal zinc-binding domain (ZBD) and C-terminal helicase domain were critical for caspase-1 inhibition. The mechanisms through which SARS-CoV-2 NSPs inhibit NLRP3 inflammasome merit further study. Altogether, early inhibition of inflammasome activation renders SARS-CoV-2 to evade host immune surveillance and may be beneficial for continued viral infection leading to enhanced pathogenicity and potential detrimental effects to the host. These results were in line with the previous report indicating reduced inflammatory responses as a predominant effect of early SARS-CoV-2 infection (72). Further investigation of the intracellular activities of viral early products would shed new light on the detailed mechanisms governing SARS-CoV-2 pathogenesis.

### 4.2 Mechanisms of inflammasome activation by SARS-CoV-2

#### 4.2.1 Disruption of intracellular ionic homeostasis

Intracellular ionic imbalance is a critical inducer of NLRP3 inflammasome activation (37). Viral proteins, which were translated during the late stage of SARS-CoV-2 infection, were able to induce NLRP3 inflammasome complex by fostering intracellular ionic disturbances (71). Intriguingly, SARS-CoV-2 E protein and ORF3a possess ion channel activity and contribute to ionic imbalances within infected cells. Calcium (Ca^2+^) flux is an important signaling event for NLRP3 inflammasome activation (73). E protein formed Ca^2+^ ion channels in ER Golgi apparatus intermediate compartment (ERGIC)/Golgi membranes and impelled reactive oxygen species (ROS)-dependent activation of NLRP3 inflammasome at the later stages of viral infection, which might contribute to advanced or complicated diseases (17, 74). It can be concluded that E protein plays a dichotomous role in NLRP3 inflammasome activation during viral infection. SARS-CoV-2 viroporin ORF3a primed NLRP3 inflammasome, inducing GSDMD activation and NF-κB-driven IL-1β expression (75). Depletion of NLRP3 abrogated ORF3a-driven caspase-1 cleavage, hinting the activation of NLRP3 inflammasome by ORF3a. NIMA-related kinase 7 (NEK7) was a critical mediator of NLRP3 assembly downstream of K^+^ efflux (76). Knockdown of NEK7 curtailed ORF3a protein’s ability to motivate NLRP3 inflammasome (75). Consistently, inhibiting K^+^ efflux obliterated ORF3a-mediated caspase-1 activation. Further study indicated that ORF3a facilitated the generation of inward-rectifier K^+^ channels in cellular membrane to drive K^+^ efflux, thereby inducing the oligomerization between NEK7 and NLRP3. This event contributed to the ignition of NLRP3 inflammasome. Similar to E protein, viral ORF3a also induced mitochondrial injury and drove mitochondrial ROS production, which potentially actuated NLRP3 inflammasome assembly and proinflammatory response (75, 77).

#### 4.2.2 Regulation of cellular proteins and signaling pathways

Human caspase-4 was shown to activate the NLRP3 inflammasome by promoting K^+^ efflux (78). A recent study indicated that SARS-CoV-2 infection induced NLRP3 activation by upregulating caspase-4 (79). The expression of caspase-4 in the lungs of COVID-19 patients showed an association with the levels of inflammasome activation markers such as caspase-1, IL-1β, IL-6 and IL-18. Deletion of caspase-11, the murine counterpart of human caspase-4, impeded disease development and reduced mortality in mice. Collectively, caspase-4 acted as a master factor mediating NLRP3 inflammasome activation and COVID-19 pathology. It is proposed that caspase-4 may be involved in viral protein-induced NLRP3 inflammasome activation. This hypothesis merits further validation. Increasing evidence indicated that SARS-CoV-2 S protein served as an inflammasome agonist (80–82). S protein selectively promoted inflammasome formation and IL-1β secretion in macrophages isolated from COVID-19 patients but not in non-COVID-19 subject-derived macrophages (83). Intriguingly, inflammasome activation was primed and the formation of IL-1β was robustly increased in macrophages from convalescent COVID-19 patients upon S protein stimulation, suggesting SARS-CoV-2-specific innate immune memory following recovery, which declined over time. Notably, S protein caused the reprogramming of human macrophages from convalescent subjects, as evidenced by the induction of inflammation- and cytokine-related pathways and inflammation/myeloid cell activation-associated epigenetic histone modifications. Such signatures and corresponding intercellular environment could permit long-lived and selective priming of the inflammasome pathway in SARS-CoV-2-infected individuals. Collectively, SARS-CoV-2 S protein acts as an ideal target against hyperinflammation in COVID-19 patients. The GU-rich single-stranded RNA (GU-rich RNA) derived from SARS-CoV-2 S protein actuated NLRP3 inflammasome and promoted the secretion of IL-1β, IL-6 and tumor necrosis factor (TNF) from human macrophages in the absence of pyroptotic cell death (84). Mechanistically, K^+^ efflux was required for GU-rich RNA-mediated NLRP3 inflammasome activation. The effect of GU-rich RNA on NLRP3 inflammasome activation also engaged the caspase-8/receptor-interacting protein kinase 1 (RIPK1)/RIPK3 signaling pathway. The TLR8/myeloid differentiation factor 88 (MyD88) pathway was necessary for SARS-CoV-2 GU-rich RNA-stimulated IL-1β maturation. Consistently, endosomal acidification blocker chloroquine prevented GU-rich RNA-induced cytokine secretion by inhibiting endosomal TLR signaling (**Table 2**). Alternative inflammasomes may contribute to cytokine burst in macrophages, which warrants additional study. It has been established that inflammasome activation and IL-1β release are not necessarily coupled to the occurrence of pyroptosis (99). Reportedly, GU-rich RNA-treated human macrophages had high expression levels of triggering receptor expressed on myeloid cells 1 (TREM1) that was essential for macrophage survival under certain circumstances (100). Human macrophages could enhance TREM1 expression to antagonize GU-rich RNA-induced pyroptosis. This may be an explanation for the enrichment of macrophages in the BALF from severe COVID-19 patients (101). IL-1 receptor-associated kinases (IRAKs) are integral to TLR-dependent activation of NLRP3 inflammasome and subsequent cytokine production (102). Pacritinib exerted inhibitory effects on TLR8-dependent inflammatory responses elicited by GU-rich RNA, contributing to the suppression of proinflammatory cytokine release from human macrophages (85). Mechanistically, pacritinib blocked IRAK1 phosphorylation and ubiquitination to suppress its association with the transforming growth factor β-activated kinase 1 (TAK1) complex, hence inhibiting TLR8-dependent NLRP3 inflammasome assembly and proinflammatory cytokine response. The anti-inflammatory activity of pacritinib in the clinical setting remains to be further studied. SARS-CoV-2 NSP6 induced NLRP3 inflammasome-mediated caspase-1 activation, IL-1β and IL-18 production, and pyroptotic cell death in lung epithelial cells (103). Mechanistically, NSP6 inhibited lysosome acidification through direct interaction with ATPase H^+^ transporting accessory protein 1 (ATP6AP1), thus resulting in autophagic flux stagnation to activate NLRP3 inflammasome. Pharmacological restoration of autophagic flux dampened NSP6-mediated NLRP3 inflammasome activation. The dysfunction of autophagic flux has been associated with the priming of AIM2 inflammasome (104). It would be rewarding to investigate the potential engagement of alternative inflammasome pathways in the pathogenicity of NSP6. The role of other viral NSPs in stimulating inflammasome activation also deserves special attention.

**Table 2:** Overview of inflammasome inhibitors and their mechanisms of action.

Drug/Chemical	Effect	Mechanism	Reference
Chloroquine	Inhibit SARS-CoV-2 GU-rich RNA-induced cytokine secretion	Block endosomal TLR signaling	(84)
Pacritinib	Inhibit SARS-CoV-2 GU-rich RNA-induced cytokine secretion	Suppress TLR8-dependent NLRP3 inflammasome assembly	(85)
25-HC@DDAB	Prevent inflammatory response and the cytokine storm	Suppress SREBP2-mediated inflammasome signaling pathway	(86)
Quercetin	Inhibit NLRP3 inflammasome activation and lessen inflammation	Reduce the expression of NLRP3, ASC and caspase-1; regulate various inflammasome regulators including NRF2, SIRT1 and TXNIP	(87)
Metformin	Inhibit NLRP3 inflammasome activation and IL-1β secretion	Restrain the formation of the NLRP3 ligand Ox-mtDNA; interfere with NLRP3 inflammasome assembly; suppress caspase-1 and GSDMD activation and IL-1β maturation; reduce the expression of ASC and caspase-1	(88, 89)
MCC950	Mitigate COVID-19-associated lung immunopathology	Inhibit NLRP3 inflammasome activation	(90)
N-acetylcysteine	Inhibit NLRP3 inflammasome activation and cytokine secretion	Block the NF-κB signaling	(91)
Niclosamide	Inhibit the activation of AIM2 and NLRP3 inflammasomes	Promote autophagy	(92)
Um-PEA	Inhibit viral S protein-induced NLRP3 inflammasome formation	–	(93)
Cannabidiol	Inhibit viral S protein-induced NLRP3 inflammasome formation	Reduce the expression of NLRP3 and caspase-1; inhibit the production of IL-1β, IL-6, IL-18 and TNF-α	(94)
Anthocyanins (C3G and P3G)	Inhibit viral S1 protein-induced secretion of IL-1β, IL-6 and IL-18	Block the NF-κB/NLRP3 inflammasome pathway	(95)
Hesperetin	Inhibit viral S1 protein-induced NLRP3 inflammasome activation	Block the Akt/MAPK/AP-1 pathway	(96)
PFEA	Inhibit viral S1 protein-induced NLRP3 inflammasome activation	Block the JAK1/STAT3 signaling	(97)
Luteolin	Inhibit viral S1 protein-induced NLRP3 inflammasome activation	Block the JAK1/STAT3 signaling	(97)
Anakinra	Inhibit IL-1 secretion and alleviate inflammation	Target IL-1 receptor	(98)

#### 4.2.3 Direct interaction with key inflammasome components

SARS-CoV-2 N protein served as an activator of NLRP3 inflammasome and could trigger excessive inflammatory responses (105). In terms of mechanism, N protein directly bound to NLRP3, fostered the interaction between NLRP3 and ASC, and primed NLRP3 inflammasome assembly. The C-terminal domain of N protein promoted its interaction between NLRP3. In addition, N protein favored the conversion of pro-IL-1β and pro-IL-6 into their active forms. As a result, N protein exacerbated lung damage and facilitated death in acute inflammation mouse models through induction of NLRP3 inflammasome activation. SARS-CoV-2 infection triggered NLRP1 inflammasome response in human lung epithelial cells (106). NLRP1 inflammasome acted as an innate immune sensor of the SARS-CoV-2 3CL protease NSP5. Specifically, NSP5 could cleave NLRP1 at the Q333 site. The repressive N-terminal fragment of NLRP1 then underwent proteasomal degradation, leading to the release of its inflammatory C-terminal fragment. This bioactive domain self-assembled and acted as a platform for caspase-1 recruitment and activation. SARS-CoV-2 NSP5-driven NLRP1 cleavage contributed to proteasome-dependent inflammasome activation. NLRP1-induced inflammation might exert a double-edged sword effect on SARS-CoV-2 immunopathogenesis. On the one hand, NLRP1 inflammasome-dependent pyroptosis restricted viral replication and potentiated alarmin/DAMP responses. On the other hand, NLRP1 inflammasome might be involved in the development of a cytokine storm. The linkage between NLRP1 inflammasome and COVID-19 immunopathology warrants further research.

Collectively, these findings suggest the existence of stage-specific mechanisms controlling inflammasome activity during viral infection. It is necessary to elucidate how SARS-CoV-2 subtly dominates NLRP3 inflammasome activation to facilitate its survival and pathogenicity. The switch molecules regulating NLRP3 inflammasome activation remain to be determined. The activation status and mechanism of other inflammasomes at different stages of SARS-CoV-2 infection are worthy of great attention. Further elucidation of the mechanisms underlying the immunoregulatory effects of viral proteins would broaden the understanding of SARS-CoV-2 immunopathogenesis.

## 5 Roles of inflammasomes in SARS-CoV-2 immunopathogenesis

**Figure 4:**
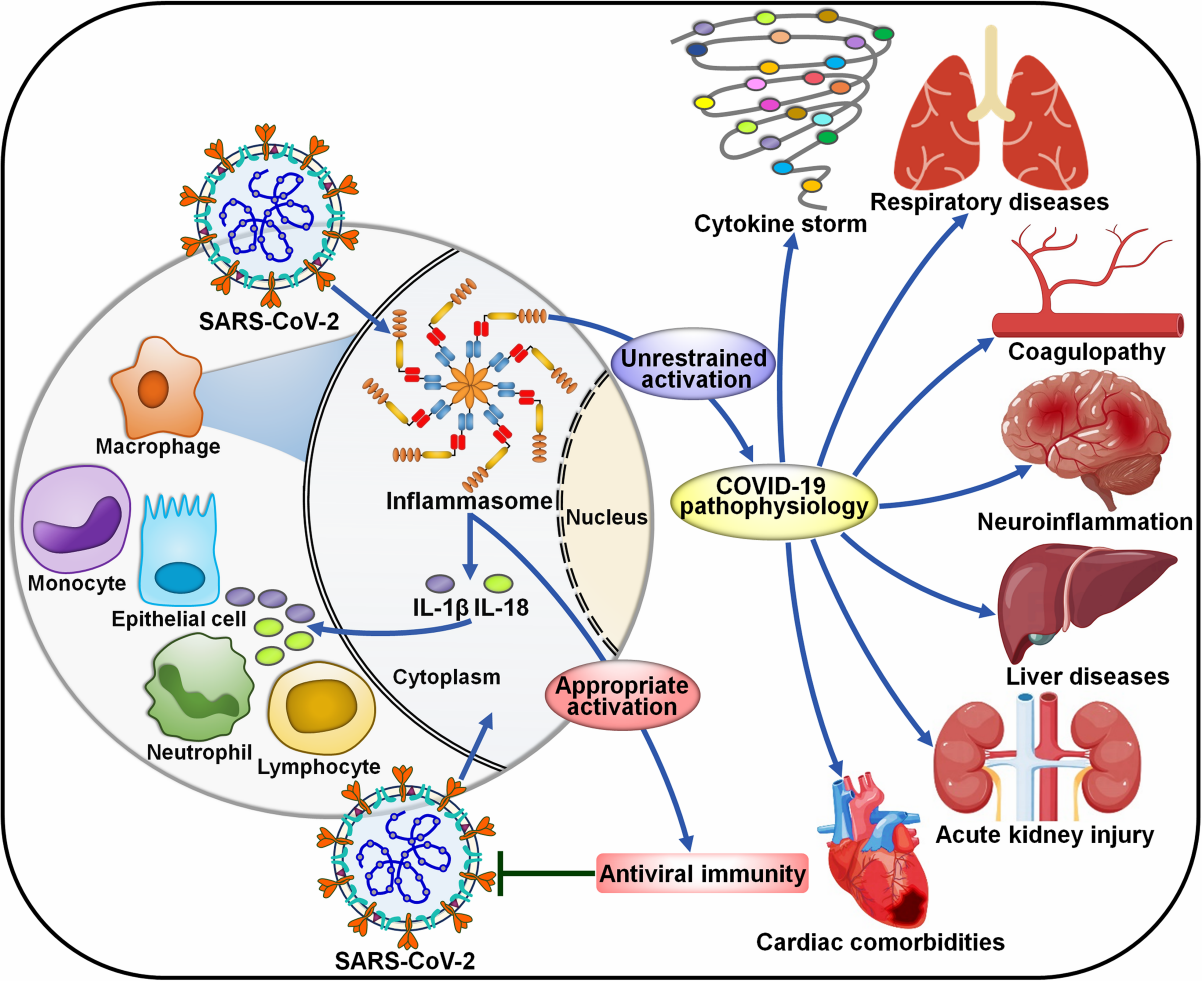
Roles of inflammasome pathways in the pathogenesis of COVID-19-associated complications. SARS-CoV-2 infection can activate inflammasomes in infected cells, especially macrophages. The inflammasome pathway constitutes a fundamental intracellular defense mechanism against pathogen invasion. However, uncontrolled activation of inflammasomes has emerged as a major driver of COVID-19-associated complications. The massive release of proinflammaotry cytokines by infected macrophages results in extensive cell damage and death, immune cell recruitment and widespread inflammasome activation, forming a proinflammatory positive feedback cascade. The over-reacted inflammasome pathway eventually leads to a cytokine storm, respiratory diseases, coagulopathy, neuroinflammation, liver diseases, acute kidney injury and cardiac comorbidities. SARS-CoV-2, severe acute respiratory syndrome coronavirus 2; IL-1β, interleukin-1β; IL-18, interleukin-18.

COVID-19 has suggested as a complex host-driven disorder that can be primarily attributable to dysregulation of host immune responses. Over-reaction of the inflammasome signaling has been associated with the pathogenesis of COVID-19-related complications, including the cytokine storm, pneumonia, ARDS, coagulopathy and neuroinflammation (**Figure 4**).

### 5.1 Cytokine storm

Cytokine secretion plays an important role in the body’s defense against viral infections, while abnormal and uncontrolled activation of host immune system can bring about serious consequences. The cytokine storm is an exuberant systemic immune response characterized by an unrestrained overproduction of proinflammatory factors such as C-X-C motif chemokine ligand 8 (CXCL8), CXCL10, interferon-γ (IFN-γ), IL-1β, IL-6, IL-18 and TNF-α (107). Available evidence has verified that COVID-19 patients had increased serum levels of proinflammatory cytokines, such as IL-1β, IL-6 and IL-10, alluding to the implication of a cytokine storm (55, 56) (**Table 3**). Patients that undergo an exacerbated cytokine response commonly present with fever, hypotension and hypoxemia (128). The syndrome can either be mitigated or progress into persistent high-grade fever and severe complications (129). As shown by previous reports, elevated levels of proinflammatory cytokines and chemokines have emerged as a major factor driving pulmonary inflammation (130). It was reported that COVID-19 patients exhibited high plasma levels of C-C motif chemokine ligand 2 (CCL2), CXCL10, IFN-γ, IL-1β, IL-7, IL-8, IL-10 and TNF-α (56). Elevated levels of CCL2, CXCL10, IFN-γ and IL-1β subsequently mounted T helper type 1 (Th1) cell responses. Remarkably, COVID-19 patients requiring intensive care unit (ICU) admission presented higher levels of CCL2, CXCL10, IL-2, IL-7, IL-10 and TNF-α than non-ICU patients, hinting the linkage between the cytokine storm and COVID-19 deterioration.

**Table 3:** Altered laboratory/clinical parameters with their clinical implications.

Author	Year	Type of study	Number of subjects	Population	Country	Results	Clinical implications	Reference
Bao et al.	2020	Meta-analysis	5912	Mild and severe COVID-19 patients	China	Increased serum levels of proinflammatory cytokines including IL-1β, IL-6 and IL-10	Cytokine storm	(55)
Huang et al.	2020	Case series	41	COVID-19 inpatients	China	Increased serum levels of proinflammatory mediators including CCl2, CXCL10, IFN-γ, IL-1β, IL-6 and IL-10 and TNF-α; enhancement of Th1 cell responses	Cytokine storm	(56)
Rodrigues et al.	2021	Case series	197	Mild, moderate and severe COVID-19 patients	Brazil	A positive correlation between the components of the NLRP3 inflammasome signaling (caspase-1 and IL-18) and inflammatory factors (CRP, IL-6 and LDH)	Disease severity	(12)
Toldo et al.	2021	Case report	8	COVID-19 patients with critical pneumonia	Italy	Aberrant activation of NLRP3 inflammasome in the lungs	Lung pathology	(108)
Wen et al.	2020	Case report	10	Recovered COVID-19 patients	China	Increased population of peripheral CD14^+^IL-1β^+^ monocytes and IFN-activated monocytes	Disease resolution	(109)
Ren et al.	2021	Case series	196	Moderate, severe and convalescent COVID-19 patients	China	Increased population of peripheral megakaryocytes and CD14^+^ monocytes	Disease severity	(58)
Szabo et al.	2021	Case series	15	Severe COVID-19 patients	The United States of America	Expanded IL-1β-expressing monocytes and macrophages in BALF	Lung pathology	(110)
Zou et al.	2020	Case series	303	Mild, moderate, severe and critical COVID-19 patients	China	High levels of D-dimers, fibrinogen and vWF, prolonged prothrombin time and low platelet number	Coagulopathy	(111)
Abernethy et al.	2020	Case report	10	COVID-19 inpatients	United Kingdom	Coexistent COVID-19 and pulmonary embolism	Coagulopathy	(112)
Demelo-Rodríguez et al.	2020	Case series	156	COVID-19 inpatients	Spain	A high incidence of deep vein thrombosis	Coagulopathy	(113)
Paul et al.	2022	Case series	19	COVID-19 ARDS patients	The United States of America	Aberrant activation of NLRP3 inflammasome; extensive microthrombi in small vessels and arterial thrombosis	Coagulopathy	(114)
Rosell et al.	2021	Case series	128	Moderate and severe COVID-19 patients	Sweden	An association between elevated levels of EV TF activity and D-dimer	Coagulopathy	(115)
Zuo et al.	2020	Case series	80	Mild and severe COVID-19 patients	The United States of America	Increased NET production by COVID-19 neutrophils	Coagulopathy	(116)
Middleton et al.	2020	Case series	50	Severe COVID-19 patients	The United States of America	Increased NET production by COVID-19 neutrophils	Coagulopathy	(117)
Skendros et al.	2020	Case series	25	Moderate, severe and critical COVID-19 patients	Greece	Increased production of TF-enriched NETs	Coagulopathy	(118)
Veras et al.	2020	Case series	32	Severe and critical COVID-19 inpatients	Brazil	Excessive NET production by COVID-19 neutrophils	Coagulopathy	(119)
Ackermann et al.	2020	Case series	24	Deceased COVID-19 patients	Germany	Widespread vascular thrombosis with microangiopathy; occlusion of alveolar capillaries	Coagulopathy	(120)
Nicolai et al.	2020	Case series	62	Moderate and severe COVID-19 patients	Germany	The existence of NET-carrying inflammatory microvascular thrombi in the heart, kidneys and lungs	Coagulopathy	(121)
Zhao et al.	2021	Case series	17	Deceased COVID-19 patients	The United States of America	Widespread microvascular thrombosis in multiple organs	Coagulopathy	(122)
Matschke et al.	2020	Case series	43	Deceased COVID-19 patients	Germany	Mild neuropathological changes with significant neuroinflammation in the brainstem	Neuropathology	(123)
Cama et al.	2021	Case report	3	Deceased COVID-19 patients	Cuba	Activation of NLRP3 inflammasome in the microglia	Neuropathology	(124)
Kroemer et al.	2020	Case series	16	Lymphopenic liver patients with COVID-19	The United States of America	Constitutive activation of NLRP3 inflammasome	Liver diseases	(125)
Ji et al.	2020	Retrospective study	202	Mild, moderate, severe and critical COVID-19 patients	China	The occurrence of persistent liver injury	Disease severity	(126)
Zhan et al.	2021	Case series	180	COVID-19 inpatients	China	Increased levels of IL-6 and IL-10	Severe liver injury	(59)
Hoel et al.	2021	Case series	55	COVID-19 inpatients	Norway	Increased markers of gut leakage and inflammasome activation including LBP, IL-18 and IL-18BP	Cardiac involvement	(127)

The inflammasome machineries are needed for the synthesis of inflammatory cytokines (73). Particularly, NLRP3 inflammasome can induce host immune responses via caspase-1-dependent pyroptosis, which results in extravasation of proinflammatory cytokines (e.g., IL-1β and IL-18) and biologically active DAMPs (131). IL-1β and IL-18 further enhance the secretion of the key cytokine IL-6 (132). The IL-6 level, a crucial mediator of acute inflammation, is deemed as a pathophysiological feature of the cytokine storm (133). It has been established that high serum IL-6 levels are positively associated with more severe clinical courses in COVID-19 patients (134, 135). IL-6 can be a predictor for COVID-19 progression (57). As expected, the components of the NLRP3 inflammasome signaling, caspase-1 and IL-18, had a positive relationship with the inflammatory factors CRP, IL-6 and LDH (12). These inflammasome-related products were also related to disease progression and patient outcome. Excessive release of proinflammatory mediators is responsible for aberrant systemic inflammatory response, which eventually results in ARDS, multiorgan failure and even death (136). Accordingly, ARDS has been considered as a deleterious outcome of the cytokine storm that can be flamed by inflammasome activation.

### 5.2 Inflammasome-induced respiratory manifestations

It was reported that the NLRP3 inflammasome was over-activated in elderly patients with COVID-19, which could be attributable to defects in mitochondrial functioning, enhanced production of mitochondrial ROS (mtROS) and massive release of mitochondrial DNA (mtDNA) (137). This caused the increased secretion of IL-1β and IL-18 in response to SARS-CoV-2 infection. Consistently, elderly patients were prone to over-react to SARS-CoV-2 infection and developed hyperinflammatory syndromes such as ARDS. Moreover, IL-1β and IL-18 have been considered as rapid inducers of lung fibrosis. Thus, over-activation of NLRP3 could be a predisposing factor contributing to high mortality in elderly patients. Compared with healthy controls, patients with fatal COVID-19 pneumonia exhibited intense expression of NLRP3 inflammasome, as manifested by increased numbers of ASC specks and high levels of NLRP3 and caspase-1 in the area of lung injury (108). These findings provided evidence linking inflammasome activation to lung pathology in COVID-19. NLRP3 is related to many inflammatory lung pathologies and abnormal activation of NLRP3 inflammasome leads to ARDS-induced pulmonary inflammation and damage (138–140). Antagonists or inhibitors of NLRP3 inflammasome could be effective in ameliorating COVID-19-related inflammation. However, the time course of NLRP3 inflammasome formation and the type of cells producing the inflammasome configuration remain to be investigated. Successive research efforts should be directed to elucidating the mechanisms of action of NLRP3 inflammasome in COVID-19 pneumonia.

The secretion of massive IL-1β into the peripheral blood enhanced the expression of other proinflammatory cytokines (e.g., IL-6), thus exaggerating SARS-CoV-2-induced cytokine storm (65). IL-1β coupling to IL-1 receptor (IL-1R) instigates the NF-κB response to enhance pro-IL-1β transcription in myeloid cells recruited to the lung tissues (141). Severe COVID-19 patients had expanded IL-1β-expressing monocytes and macrophages in BALF and increased population of peripheral CD14^+^IL-1β^+^ monocytes and macrophages (58, 109, 110). Inflammasome activation and subsequent cytokine response can be amplified through positive feedback loops, leading to the creation of a hyperinflammatory environment comprising multitudinous cytokine-producing immune cells associated with severe COVID-19 (142). Intense immune cell infiltration and cytokine secretion in lung tissues are representative features of ARDS and may ultimately lead to severe lung injury in COVID-19 patients. The cytokine storm may also form a positive feedforward loop to escalate lung damage. IL-1β-induced activation of endothelial cells inhibited the transcription of vascular endothelial cadherin (VE-cadherin), contributing to the disruption of vascular integrity (143). IL-1β-induced IL-6 release enhanced the formation of vascular endothelial growth factor (VEGF) that damaged the pulmonary endothelium via VE-cadherin internalization (144). This event facilitated accumulation of interstitial and alveolar fluid that compromised pulmonary gas exchange (145, 146). Because of the reduction of resorptive activity from infected alveolar epithelium, alveolar fluid accumulation could result in a series of unfavorable consequences such as disturbance of pulmonary surfactant, and alveolar instability and breakdown (147, 148). Immune cell recruitment and tissue failure could cooperatively exaggerate lung damage in response to aberrant release of IL-1-directed proinflammatory cytokines.

### 5.3 Inflammasome-directed coagulopathy

SARS-CoV-2 infection causes inflammation-directed coagulopathy, which may account for venous/arterial thrombosis and unfavorable outcomes in COVID-19 patients (**Figure 4**). A previous study demonstrated that a majority of COVID-19 patients had coagulation aberrations that manifested as high levels of D-dimers, fibrinogen and von Willebrand factor (vWF), prolonged prothrombin time and low platelet number (111). Thrombi have been described in the brain, heart, lungs, liver and kidneys of COVID-19 patients (149). The degree of thrombotic complications was heightened with increasing illness severity (150, 151). Severe cases of COVID-19 presented with deep vein thrombosis, pulmonary embolism and disseminated intravascular coagulation (112, 113). Inflammasomes have emerged as part of the mechanism responsible for SARS-CoV-2-induced thrombosis due to their regulatory effects on clot retraction and arterial thrombosis (152). Compared with non-COVID-19 subjects, the expression of NLRP3 and caspase-1 was increased along the vascular wall in COVID-19 ARDS cases, suggesting the activation of NLRP3 inflammasome in vessel wall (114). Vascular alternations including arterial thrombosis, microthrombi in small vessels and organization were widespread in lungs from COVID-19 patients compared with healthy controls. After viral entry, SARS-CoV-2 attaches to the alveolar epithelium. The infected lung cells then release cytokines and chemokines, which recruit neutrophils to the alveolar space. These events may contribute to the collapse of the alveolar wall. Moreover, the NLRP3 inflammasome was also activated in endothelial cells in response to various stimuli (e.g., SARS-CoV-2 infection, cytokines or chemokines), leading to endothelial pyroptosis. As a result, the endothelial-alveolar barrier was destroyed accompanied by the flooding of interstitial and alveolar spaces. Endothelial cell death and debris act as an inducer of coagulation cascades that foster thrombi formation.

Inflammasome activation in monocytes and neutrophils propels coagulation via distinct mechanisms. Inflammasome-mediated pyroptosis causes extensive coagulopathy independent of cytokine release. Previous studies suggested that induction of the inflammasome pathway contributed to systemic blood clotting and extensive thrombosis in tissues, and GSDMD but not proinflammatory cytokine was required for these coagulation cascades. Following inflammasome formation, GSDMD-executed pyroptosis of macrophages promoted the release of extracellular vesicles carrying high levels of membrane-bound tissue factor (TF) that subsequently entered the circulation and drove coagulation through activation of prothrombin (153). As expected, COVID-19 patients had elevated levels of circulating TF-positive extracellular vesicles, which correlated with disease severity and outcome (115). In addition, GSDMD pores that were formed downstream of inflammasome activation acted as the conduit for Ca^2+^ influx. This activated the phospholipid scramblase transmembrane protein 16F (TMEM16F), which increased TF activity through phosphatidylserine externalization (154).

Extracellular traps (NETs), extracellular DNA fibers decorated with antimicrobial proteins, are extruded from activated neutrophils (155). NETs are responsible for ensnaring and immobilizing extracellular pathogens and form an effector mechanism of host innate immune defense (156). Dysregulated NET formation during the course of COVID-19 can be a trigger of inflammation, blood coagulation and immunothrombosis (116). As NLRP3 inflammasome induced the rupture of nuclear envelope and cellular membrane, it was needed for NET protruding from neutrophil membrane (157, 158). It is conceivable that inflammasomes play an important role in COVID-19 coagulopathy. Reportedly, the circulating neutrophils of COVID-19 patients displayed elevated activation of NETs (117–119). Extensive neutrophil infiltration and abundant NETs in the lungs have been described in COVID-19 patients, which may trigger the occlusion of the pulmonary vasculature (120, 159, 160). Importantly, increased levels of NET-producing neutrophils positively correlated with the risk of morbid thrombotic complications in COVID-19 patients (116). NET-induced endothelial microvascular thrombosis was likely an explanation for thrombosis in COVID-19 (161). NET-released histones and cytotoxic enzymes caused endothelial cell death and endothelial dysfunction, culminating in organ damage in COVID-19 patients (162, 163). NETs induced acute lung injury in COVID-19 patients by accelerating lung epithelial cell death and intravascular thrombus synthesis (164, 165). In addition, NET-carrying inflammatory microvascular thrombi were detected in the heart, kidneys and lungs of COVID-19 patients (121). The pro-thrombiotic effect of NETs might be a cause of organ injury in COVID-19 patients (122). The inflammasome pathway could contribute to COVID-19 thrombus by regulating NET formation (116, 166). There is a great need for more research into the pathogenic mechanisms underpinning inflammasome-mediated immunothrombosis in COVID-19. Therapeutic interventions that aim to suppress NET formation by neutrophils will be necessary to prevent immunothrombosis and organ failure in COVID-19 patients.

### 5.4 Inflammasome-mediated neuroinflammation

SARS-CoV-2 RNA or proteins could be detected in many typical cells of central nervous system (CNS) (123, 124). Importantly, NLRP3 inflammasome was activated in monocytes or macrophages from lungs and brain, suggesting the important role of NLRP3 inflammasome in SARS-CoV-2-induced injuries of both the respiratory and nervous systems. Neurotropism of SARS-CoV-2 might foster blood-brain barrier leakage, leading to the impairment of the CNS vascular endothelium. The receptors angiotensin-converting enzyme 2 (ACE2) and cluster of differentiation 147 (CD147) mediated SARS-CoV-2 entry into cells of neuronal origin (e.g., astrocytes, microglia and neurons) (167, 168). Astrocytes and microglia are two main sources of proinflammatory cytokines and are critical for SARS-CoV-2-induced neuroinflammation (169). SARS-CoV-2 infection initiated robust NLRP3 inflammasome activation in microglia (170). Moreover, SARS-CoV-2 S protein primed inflammasome assembly in response to diverse stimuli (e.g., ATP and nigericin) via the NF-κB signaling pathway. Depletion of NLRP3 dampened viral S protein-induced inflammasome activation. SARS-CoV-2 S protein subunit 1 (S1) could elevate the expression level of NLRP3 inflammasome and increased caspase-1 activity in microglia (171). Moreover, S1 increased the production of IL-1β, IL-6 and TNF-α that were hallmarks of neuroinflammation, suggesting the role of S protein in the pathophysiology of neurological diseases. Knowledge of inflammasome-mediated neuroinflammation during SARS-CoV-2 infection is warranted to dissect the pathogenic mechanisms of neurological disorders in COVID-19 patients. Furthermore, S1 was able to stimulate the NF-κB and p38 mitogen-activated protein kinase (MAPK) signaling pathways through its regulatory action on TLR4 (171). Inhibition of p38 could block S1-mediated secretion of IL-6 and TNF-α, implying that the p38 MAPK pathway was implicated in S1-induced hyperinflammation. Reportedly, NF-κB triggered NLRP3 through caspase-1 (172). It is equivocal whether S1-mediated NLRP3 inflammasome activation is ascribed to its direct influence on NLRP3 activity or an indirect effect as a consequence of NF-κB activation. The contribution of NLRP3 inflammasome to S1-mediated neuroinflammation needs thorough investigation. Similarly, SARS-CoV-2 infection caused the priming of NLRP3 inflammasome and induced caspase-1-mediated secretion of IL-1β and IL-18 from human astrocytes (173). The inflammasome-derived product caspase-1 could facilitate neuroinflammation by interrupting the blood-brain barrier (174). IL-1β and IL-18 facilitated the production of other proinflammatory cytokines by astrocytes, microglia and neurons (175). Proinflammatory cytokines increased the permeability of the blood-brain barrier, activated astrocytes and microglia, and eventually promoted the infiltration of immune cells into the CNS (176). In addition, activated astrocytes and microglia triggered the release of cytokines and chemokines, including CCL2, CXCL10, IL-6 and TNF-α (177). IL-18 enhanced caspase-1 activity and potentiated proinflammatory cytokine responses in the CNS (178). Such pathological processes further exaggerated neuroinflammation-induced neuronal damage and led to neurological presentations in COVID-19 patients. Altogether, these studies provide scientific-based evidence to support hidden mechanisms underlying innate immune activation following SARS-CoV-2 infection in the CNS and open up new opportunities for developing new therapeutic avenues to relieve SARS-CoV-2-induced neurological symptoms.

### 5.5 Inflammasome activation and other pathologies

The NLRP3 inflammasome was constitutively activated in COVID-19 patients with underlying liver diseases such as cirrhosis, acute live failure and non-alcoholic fatty liver disease (NAFLD) (125). SARS-CoV-2 infection further motivated NLRP3 inflammasome, as evidenced by increased caspase-1 activity in T cells and high levels of IL-18 and LDH. The inflammasome-dependent pyroptosis of T cells might not only cause defective adaptive immunity but also induce lethal inflammatory responses through extravasation of proinflammatory factors. It is likely that extravagant inflammasome activity exacerbates inflammation and immune dysfunction, ultimately leading to severe illnesses in patients with liver diseases. Collectively, inflammasome activation is a significant contributing factor to poor COVID-19 outcomes in comorbid patients. Liver injury was observed in COVID-19 patients (126). Increased levels of IL-6 and IL-10 were associated with a high risk of severe liver damage in COVID-19 patients (59). The over-activation of inflammasomes induces excessive cytokine release by immune cells, which can trigger a systematic inflammatory cytokine storm, leading to liver injury in infected individuals (179). Further research is still required to disclose the exact mechanisms of COVID-19-associated liver injury. Acute kidney injury (AKI) is a critical issue in COVID-19 patients (180). Severe COVID-19 patients that developed AKI had increased levels of proinflammatory cytokines (e.g., IFN-γ, IL-1β, IL-8 and TNF-α) compared with non-severe patients (181). Inflammasomes are likely an underlying contributor to hyperactivated inflammatory response and vascular thrombosis that consequently lead to AKI in COVID-19 patients (182). Moreover, the cytokine storm may cause renal impairment by triggering intrarenal inflammation (183). However, whether the inflammasome pathway is an important cause of SARS-CoV-2-associated AKI has yet to be examined.

SARS-CoV-2 infection has been connected with various cardiovascular diseases such as acute coronary syndrome, myocardial injury, arrhythmia and myocarditis (184). Inflammasome activation has been documented in COVID-19 patients with cardiac involvement, as shown by increased plasma levels of inflammasome markers including IL-1β, IL-1R antagonist (IL-1RA), IL-18 and IL-18-binding protein (IL-18BP) (127). NLRP3 inflammasome triggered hyperinflammatory responses by intensifying COVID-19-associated inflammation or coordinating the ACE2/angiotensin signaling cascade (185). Over-inflammation likely facilitates viral infiltration of other organs including the cardiovascular system. Cardiac cells with high expression of ACE2 can be a direct target of SARS-CoV-2 infection. Viral S protein-mediated ACE2 dysregulation in cardiac cells triggered NLRP3 inflammasome activation and proinflammatory cytokine response, hence aggravating cardiovascular disease comorbidity in COVID-19 (186). Meanwhile, ACE2-relevant signaling pathway may contribute to myocardial injury. Excessive cytokine release downstream of inflammasome priming gave rise to an extremely high demand for metabolites and energy, thus exacerbating myocardial damage (186). IL-1RA, IL-18 and IL-18BP were intertwined with increased levels of troponin, suggesting that inflammasome activation had a relationship with myocardial injury (127). The levels of IL-1, IL-6 and TNF-α were positively associated with increased risk of arrhythmia in COVID-19 patients (187). The mechanism underlying inflammasome activation in COVID-19 patients with cardiac involvement needs to be revealed. Unfavorable cardiovascular disorders following viral infection can be mainly attributable to inflammasome action, but it is still essential to define the mechanistic linkage between inflammasome activation and the development of cardiac comorbidities in COVID-19 patients.

It is increasingly acknowledged that host genetic variation is a crucial factor affecting COVID-19 severity (188–190). The genome-wide association meta-analyses that consisted of 49,562 COVID-19 patients from 46 studies across 19 countries indicated that specific mutations of the tyrosine kinase 2 (*TYK2*) gene could enhance the risk of becoming critically ill with COVID-19, while individuals who carried a variant in the dipeptidyl peptidase 9 (*DPP9*) gene were at elevated risk of developing critical disease (191, 192). Loss-of-function mutations in type I IFN (IFN-I) immunity genes (*TLR3* and *TLR7*) might be connected with severe forms of COVID-19 in European populations (193–195). NLRP3 inflammasome gene variants were correlated with critical COVID-19 in the Brazilian population (196). The genome-wide association study among the Chinese population showed that the most pronounced gene variant related to COVID-19 severity was located in TMEM189-UBE2V1 that was implicated in the IL-1 signaling cascade (197). The variability in the frequency of inflammasome-relevant gene variants among individuals from other ancestry groups remains to be comprehensively evaluated. The identified host genetic factors are mainly intertwined with essential pathophysiological processes, including immune responses and inflammation (198). It is worth noting that these associations must be interpreted with caution and need to be further verified. Future studies including genetically diverse study groups will be required to identify the genomic loci associated with COVID-19 outcomes. A thorough investigation of COVID-19 host genetics will provide new insight into pathogenesis, risk stratification, therapy response as well as precision medicine.

## 6 Therapeutic potential of inflammasome-targeting interventions in COVID-19

### 6.1 Preclinical studies

**Figure 5:**
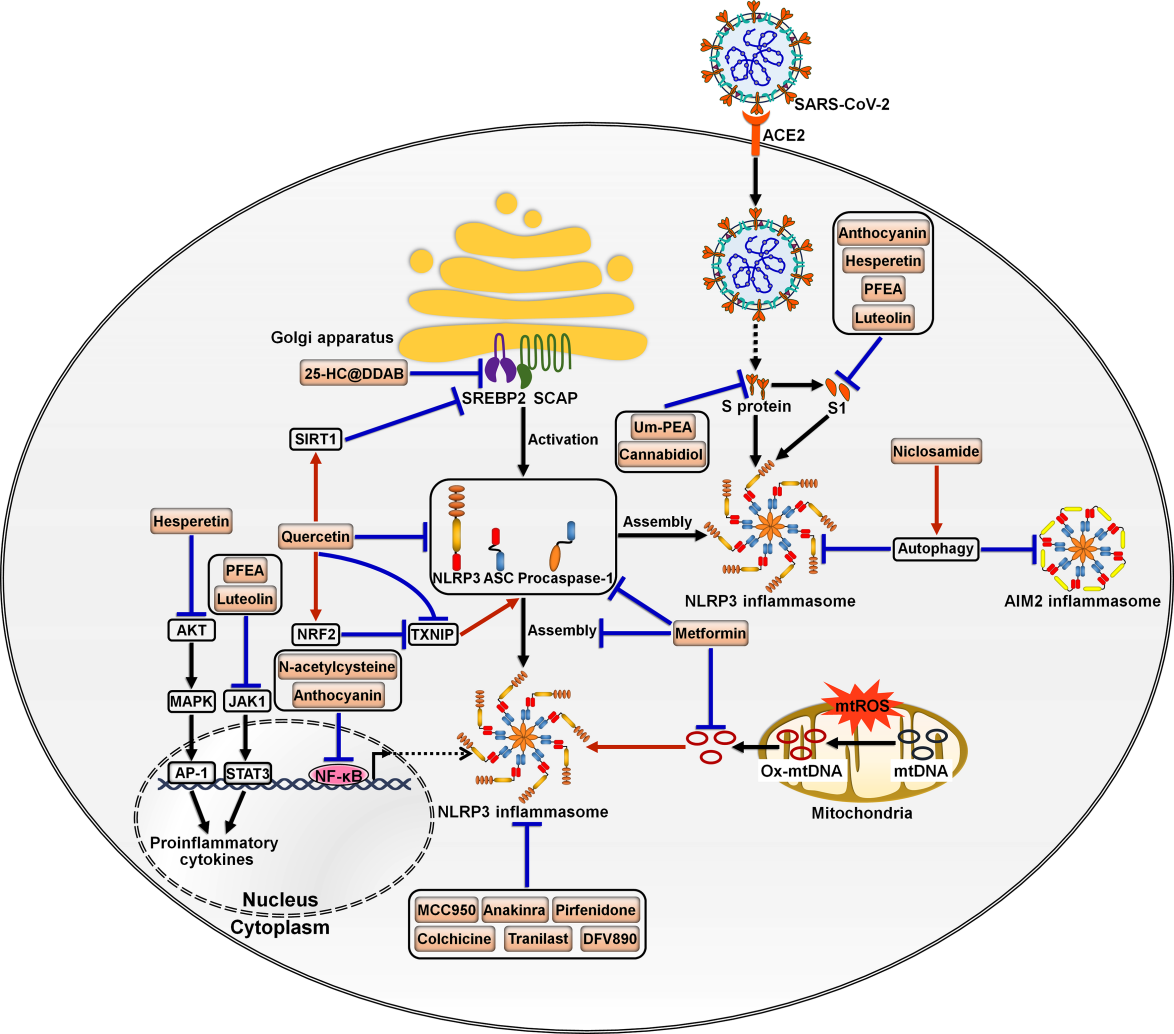
Potential inflammasome-targeted therapies for COVID-19. Various chemicals have an inhibitory effect on inflammasome activation and hold great promise in the treatment of COVID-19-associated complications. 25-HC@DDAB suppresses SREBP2-mediated inflammasome signaling pathway. Um-PEA and cannabidiol can abrogate SARS-CoV-2 S protein-stimulated NLRP3 inflammasome formation. Anthocyanin inhibits viral S1-induced inflammatory responses by targeting the NF-κB/NLRP3 inflammasome pathway. Likewise, N-acetylcysteine impedes SARS-CoV-2-induced inflammasome activation and cytokine secretion by targeting NF-κB. Hesperetin represses S1-induced NLRP3 inflammasome activation by blocking the Akt/MAPK/AP-1 signaling pathway. PFEA and luteolin restrain S1-driven proinflammatory response by downregulating the JAK1/STAT3 signaling cascade. Niclosamide restricts the activation of AIM2 and NLRP3 inflammasomes, which can be partially ascribed to its pro-autophagic property. Quercetin reduces the production of NLRP3 inflammasome components such as NLRP3, ASC and caspase-1 and coordinates inflammasome regulators SIRT1, NRF2 and TXNIP. Metformin prevents the formation of the NLRP3 ligand Ox-mtDNA, leading to the inhibition of NLRP3 inflammasome activation. Moreover, metformin decreases the expression of NLRP3 inflammasome components ASC and caspase-1 and inhibits the assembly of NLRP3 inflammasome. MCC950 and anakinra also act as NLRP3 inflammasome inhibitors and may be of potential translational value in severe COVID-19 patients. Some inflammasome antagonists including pirfenidone, colchicine, Tranilast and DFV890 are under development and being explored for their therapeutic efficacy in COVID-19 patients. SARS-CoV-2, severe acute respiratory syndrome coronavirus 2; ACE2, angiotensin-converting enzyme 2; SREBP2, sterol regulatory element-binding protein 2; SCAP, SREBP cleavage-activating protein; S protein, spike protein; S1, S protein subunit 1; SIRT1, sirtuin 1; NLRP3, nucleotide-binding oligomerization domain-like receptor family pyrin domain-containing protein 3; ASC, apoptosis-associated speck-like protein containing a caspase recruitment domain; AIM2, absent in melanoma 2; AKT, protein kinase B; NRF2, nuclear factor erythroid 2-related factor 2; TXNIP, thioredoxin-interacting protein; MAPK, mitogen-activated protein kinase; JAK1, Janus kinase 1; mtROS, mitochondrial reactive oxygen species; mtDNA, mitochondrial DNA; Ox-mtDNA, oxidized-mitochondrial DNA; AP-1, activator protein-1; STAT3, signal transducer and activator of transcription 3; NF-κB, nuclear factor-κB.

Dysregulation of the inflammasome signalings could result in tissue and organ impairment in various systems of the human body (199). Harnessing the inflammasome to restrict the proinflammatory milieu may provide novel therapeutic modalities for COVID-19. So far, a multitude of inhibitors have been introduced for the key components of the inflammasome pathways (**Figure 5**). Kim et al. (86) developed 25-hydroxycholesterol (25-HC) and didodecyldimethylammonium bromide (DDAB) nanovesicles, namely 25-HC@DDAB, as an anti-COVID-19 drug candidate (**Table 2**). The *in vivo* study indicated that 25-HC@DDAB showed preferential accumulation to the lung tissues. Particularly, 25-HC@DDAB suppressed sterol regulatory element-binding protein 2 (SREBP2)-mediated inflammasome signaling pathway in PBMCs isolated from COVID-19 patients, hence ameliorating excessive inflammatory response and the cytokine storm. Accordingly, the nanotherapeutics may be an effective therapeutic approach for COVID-19 treatment. However, further clinical studies are necessary to comprehensively evaluate the safety and therapeutic efficiency of 25-HC@DDAB. Quercetin was able to restrict the production of NLRP3 inflammasome components such as NLRP3, ASC and caspase-1 and coordinate various inflammasome regulators (e.g., NRF2, SIRT1 and TXNIP) (87). Thus, quercetin acted as an NLRP3 inflammasome inhibitor and could be exploited to dampen severe inflammation in COVID-19 patients. Metformin was reported to repress NLRP3 inflammasome activation and IL-1β generation in macrophages (88). Mechanistically, metformin could restrain new mtDNA synthesis, which was essential for the formation of the NLRP3 ligand oxidized (Ox)-mtDNA, leading to the inhibition of NLRP3 inflammasome activation. Moreover, metformin exerted direct effects on NLRP3 inflammasome. Metformin affected the assembly of NLRP3 inflammasome instead of the expression of its components. Furthermore, metformin negatively modulated caspase-1 and GSDMD activation and IL-1β maturation. Administration of metformin reduced SARS-CoV-2-induced pulmonary inflammation and ARDS in SARS-CoV-2 infectable hACE2 transgenic mice. Another study demonstrated that melatonin treatment was able to restrain NLRP3 inflammasome priming by downregulating ASC and caspase-1 (89). It also inhibited the production of IL-1β and TNF-α in COVID-19 patients. Melatonin may represent an attractive supplemental treatment to alleviate immune over-activation and the cytokine storm in COVID-19 patients. Continued research efforts are needed to assess the therapeutic efficacy of metformin in COVID-19 patients.

SARS-CoV-2 infection triggered NLRP3 inflammasome activation and increased the levels of caspase-1, IL-1β and IL-18 in the lung tissues, which precipitated systemic inflammation and exacerbated lung immunopathology *in vivo* (60, 90). Blockade of NLRP3 inflammasome via genetic knockout and specific inhibitor MCC950 apparently restrained NLRP3 inflammasome activation induced by SARS-CoV-2 infection (90). Furthermore, MCC950 abated COVID-19-associated lung immunopathology in SARS-CoV-2-infected mice, as evidenced by fewer signs of interstitial pneumonia, reduced accumulation of immune cells (e.g., macrophages and neutrophils) and decreased levels of proinflammatory mediators (e.g., IL-1β, IL-6, IL-18, TNF-α, monocyte chemoattractant protein-1 (MCP-1), CCL2 and CXCL9) in lung tissues. It is proposed that NLRP3 is directly involved in production of SARS-CoV-2 replication complex. Activation of NLRP3 inflammasome may also contribute to the formation of a favorable environment for viral replication. The implication of NLRP3 inflammasome in SARS-CoV-2 replication awaits follow-up research. NLRP3 inflammasome favors SARS-CoV-2 propagation, leading to high viral loads and increasing infectiousness, which results in direct pulmonary injury. Moreover, SARS-CoV-2 can trigger a systematic cytokine storm that impels the progression of more advanced diseases in COVID-19 patients (200). Over-activation of NLRP3 inflammasome has been suggested as a pathological event responsible for the formation of SARS-CoV-2-induced cytokine storm (201). Targeting NLRP3 inflammasome by MCC950 could be a promising immunotherapeutic approach for treating COVID-19. NLRP3 inflammasome was over-activated in airway tissues from severe COVID-19 patients evidenced by increased levels of the inflammasome components including NLRP3, ASC and caspase-1 and massive production of IL-1β and IL-18 (91). The activation of NLRP3 inflammasome was connected with systemic inflammation and mortality. Mechanistic investigation showed that SARS-CoV-2 proteins primed NLRP3 inflammasome in human macrophages and bronchial epithelial cells by activating the TLR2/MyD88/NF-κB axis. N-acetylcysteine inhibited SARS-CoV-2-induced inflammasome activation and cytokine secretion by targeting NF-κB. N-acetylcysteine may have the potential to curb hyperinflammation and the cytokine storm in COVID-19 patients, mandating further study of the therapeutic efficacy and mechanism of N-acetylcysteine. Niclosamide effectively repressed the activation of AIM2 and NLRP3 inflammasomes, which was partially ascribed to its pro-autophagic property (92). It was worth noting that diverse mechanisms might operate in niclosamide-mediated suppression of inflammasome activation. The pathways that contribute to its anti-inflammatory action need to be further studied. Whether niclosamide is effective in preventing inflammation in COVID-19 patients is a vital future question to pursue.

SARS-CoV-2 S protein caused NLRP3 inflammasome activation and the extravasation of proinflammatory cytokines (IL-1β and IL-6) by upregulating NLRP3 and caspase-1 (93). Ultramicronized palmitoylethanolamide (um-PEA) abrogated S protein-stimulated NLRP3 inflammasome formation. Therefore, um-PEA held promise in the fight against COVID-19. Similarly, cannabidiol (CBD) had the ability to resist excess inflammatory response induced by S protein (94). Mechanistically, CBD was capable of downregulating the expression of NLRP3 and caspase-1 and preventing the production of IL-1β, IL-6, IL-18 and TNF-α. Thus, CBD functioned as an inhibitor of SARS-CoV-2 S protein. It is worth noting that the aforementioned conclusions were drawn from *in vitro* experimental results. Further *in vivo* and clinical studies are critically needed to substantiate the protective potential of um-PEA and CBD in COVID-19 patients. Active anthocyanins (C3G and P3G) derived from black rice germ and bran (BR extract) could reduce SARS-CoV-2 S1-induced secretion of IL-1β, IL-6 and IL-18 in macrophages by targeting the NF-κB/NLRP3 inflammasome pathway (95). C3G and P3G exerted anti-inflammatory effects and could be developed into preventive agents for SARS-CoV-2-induced inflammation. Continual efforts should be made to identify the upstream molecules (e.g., TLR and MyD88) involved in the anti-inflammatory actions of C3G and P3G. The health benefits of these anthocyanins should be confirmed in COVID-19 patients. Hesperetin from root extract of *Clerodendrum petasites* S. Moore decreased SARS-CoV-2 S1-induced expression of NLRP3, ASC and caspase-1 through counteraction with the protein kinase B (Akt)/MAPK/activator protein-1 (AP-1) pathway (96). Hesperetin markedly attenuated the production of proinflammatory cytokines including IL-1β and IL-8. These findings provided evidence for the potential application of hesperetin in the prevention of SARS-CoV-2-induced chronic inflammation. The ethyl acetate fraction of *Perilla frutescens* (PFEA) and luteolin were found to quench SARS-CoV-2 S1-induced NLRP3 inflammasome activation in lung cells via downregulation of the Janus kinase 1 (JAK1)/signal transducer and activator of transcription 3 (STAT3) signaling, as shown by decreased expression of NLRP3, ASC and caspase-1 (97). PFEA and luteolin could be introduced as preventive agents against inflammation-associated post-acute sequelae in COVID-19 patients. Consistent with previous studies, activated NLRP3 inflammasome, accompanied by IL-1β release, was observed in circulating monocytes from severe COVID-19 patients (98). The inflammasome activation was retarded by administration of the IL-1 receptor antagonist anakinra, which might be of potential translational value in severe COVID-19 patients.

### 6.2 Clinical studies

Some inflammasome inhibitors are under development and being explored for their therapeutic benefits in COVID-19 intervention (**Table 4**). Pirfenidone, an approved drug for the treatment of idiopathic pulmonary fibrosis, could inhibit the assembly of NLRP3 inflammasome and alleviate lung inflammation and the duration of hospital stay in COVID-19 patients (202). Inhibition of NLRP3 inflammasome by pirfenidone may be a prospective therapeutic option for COVID-19. Colchicine, an alkaloid derived from *Colchicum autumnale*, can inhibit NLRP3 inflammasome activation (209, 210). Colchicine may be used as a mediation that acts on NLRP3 infammasome-mediated cytokine storm in COVID-19. The therapeutic potential of colchicine was previously investigated in nine COVID-19 patients (204). The results indicated that colchicine was well tolerated and effective in lessening fever. A randomized controlled clinical study including 105 COVID-19 patients showed that colchicine decreased the clinical deterioration rate. In addition, colchicine could reduce the duration of hospital stay in COVID-19 patients (205). Given the great potential of colchicine to reduce COVID-19-associated inflammation, the efficacy of colchicine plus the IL-6 receptor antagonist (IL-6Ra) tocilizumab is being evaluated in an open-label randomized controlled trial (206). Larger randomized controlled clinical trials will be necessary to corroborate the beneficial effects of colchicine in COVID-19 treatment. Of note, a minority of patients treated with colchicine developed gastrointestinal symptoms such as diarrhoea, nausea or vomit. The adverse effects relevant to the intake of colchicine are correlated with prolonged use, pre-existing diseases and potential interaction with other agents. Thus, the safety and side effects of colchicine are worthy of detailed investigation. In a randomized controlled trial, Tranilast, a potential NLRP3 inflammasome inhibitor, was used as a complementary treatment in combination with standard therapeutic measures (e.g., antivirals and supportive care) in COVID-19 patients (207). Tranilast treatment could ameliorate clinical symptoms including weakness and fatigue and reduce hospital stay. COVID-19 patients treated with Tranilast had apparently lower levels of proinflammatory cytokines (e.g., IL-1β, IL-6 and TNF-α) than the controls. Furthermore, the results of the quantitative outcome of blood oxygen saturation showed that Tranilast treatment produced significant effectiveness. Collectively, Tranilast could improve the clinical outcome of COVID-19 patients. Nevertheless, more clinical studies are warranted to assess the protective potency of Tranilast. It would be equally important to illuminate the mechanisms contributing to the therapeutic effect of Tranilast. An early phase 2a randomized clinical trial including 143 patients with COVID-19 pneumonia and impaired respiratory function was previously conducted to assess the clinical efficacy of the NLRP3 inhibitor DFV890 (208). As a result, DFV890 was well-tolerated and had an acceptable safety profile. Patients treated with DFV890 showed early viral clearance, improved clinical status and in-hospital outcomes and decreased mortality rate compared with the standard-of-care group. These results provided evidence to support the utilization of DFV890 as a complementary therapy for COVID-19-related ARDS. Collectively, these studies suggest that inflammasome-based therapy holds great promise in the treatment of COVID-19-associated complications. However, some limitations such as the small sample size and the short duration of follow-up may increase the probability of bias. Well-designed multi-center trials with a large sample size will be able to verify existing clinical results in the future.

**Table 4:** Clinical trials of inflammasome-targeted therapies in COVID-19.

Drug candidate	Clinical trial	Sample size	Population	Country	Mechanism of action	Outcome	Reference
Pirfenidone	Phase 3 clinical trial	146	Male and non-pregnant female COVID-19 patients	China	Inhibit NLRP3 inflammasome assembly	Reduce lung inflammation and the duration of hospital stay	(202)
Colchicine	Open-label, randomized clinical trial	105	COVID-19 inpatients	Greece	Inhibit NLRP3 inflammasome activation	Alleviate the clinical deterioration rate	(203)
Colchicine	Randomized clinical trial	9	Domiciliary consecutive COVID-19 patients	Italy	Inhibit NLRP3 inflammasome activation	Lessen fever	(204)
Colchicine	Randomized controlled trials	17,976	COVID-19 patients	Global, Brazil, Colombia, Greece, Iran, Russia, Spain, United Kingdom	Inhibit NLRP3 inflammasome activation	Reduce the duration of hospital stay	(205)
Colchicine/tocilizumab	Open-label, randomized clinical trial	~230	Severe COVID-19 patients	Qatar	Inhibit NLRP3 inflammasome activation and retard IL-6 secrection	Unpublished	(206)
Tranilast	Open-label, randomized controlled clinical trial	72	Severe COVID-19 patients	Iran	Reduce the levels of proinflammatory cytokines including IL-1β, IL-6 and TNF-α	Ameliorate clinical symptoms including weakness and fatigue; reduce hospital stay; increase blood oxygen saturation	(207)
DFV890	Phase 2, randomized, controlled, open-label, multicentre study	143	Hospitalized COVID-19 patients with impaired respiratory function	Argentina, Brazil, Denmark, Germany, Hungary, India, Mexico, Netherlands, Peru, Russia, South Africa, Spain	Inhibit NLRP3 inflammasome activation	Promote viral clearance; improve clinical status and in-hospital outcomes; decrease mortality rate	(208)

## 7 Conclusion and future perspectives

The formation and activation of inflammasomes are crucial for counteracting pathogen invasion. However, unrestrained activation of inflammasomes may cause a cytokine storm, systemic hypercoagulation, ARDS and even death. Over-exuberant inflammasome response has emerged as an important predisposing factor for disease severity and poor clinical outcome in COVID-19 patients. So far, NLRP3 is the most extensively studied inflammasome in COVID-19 owing to its critical role in mediating infection-induced inflammation. SARS-CoV-2 plays opposite roles in controlling NLRP3 inflammasome function. SARS-CoV-2 infection induces the activation of NLRP3 inflammasome that triggers antiviral immune response. *In vivo* studies are expected to be carried out to determine the protective role of the inflammasome signaling during the early stage of SARS-CoV-2 infection. SARS-CoV-2 have evolved multiple mechanisms to antagonize the priming of NLRP3 inflammasome. On the contrary, the virus can fuel unbridled inflammatory reaction by triggering NLRP3 inflammasome. As there is relatively little literature available on this topic, our understanding of the intricate interconnection between SARS-CoV-2 and NLRP3 inflammasome is still incomplete. It is of value to figure out how SARS-CoV-2 takes advantage of NLRP3 inflammasome to ensure its own survival and pathogenesis. NLRP1, a sensor for RNA virus infection, was found to be activated in SARS-CoV-2-infected subjects (106, 211). It is proposed that AIM2 inflammasome plays a fundamental role in antiviral response against SARS-CoV-2 infection (63). SARS-CoV-2 infection could facilitate nutrient deficiency and induce excessive cell death, contributing to the liberation of endogenous DNA. Moreover, SARS-CoV-2 invasion may cause the release of mtDNA into the cytoplasm by impairing mitochondrial membranes. Both free DNA and mtDNA could act as activators of AIM2 inflammasome. However, proinflammatory products resulting from NLRP1 and AIM2 inflammasomes may overlap with those of NLRP3 inflammasome activation. Thus, it is not surprising that previous studies do not center on the interrelation between these inflammasomes and COVID-19. Considerably more work will be needed to investigate the potential engagement of other inflammasomes in COVID-19 and unravel the mechanisms associated with their activation and synergistic functions. It appears that the lung tissues secrete inflammasome component-carrying extracellular vesicles into the circulation following SARS-CoV-2 infection, leading to non-respiratory manifestations. More evidence is required to determine whether extracellular vesicles act as a key mediator of SARS-CoV-2-induced pathologies. It is worth noting that the prolonged activation of inflammasomes does not always cause hyperinflammation in some COVID-19 patients (212). The discrepancy may be ascribed to the duration or magnitude of inflammasome-activating insults, the location of inflammation or the type of cells that are inflamed. There may exist negative feedback mechanisms that hinder the inflammasome signaling. Continual efforts must be made to address the variability in inflammasome-induced immunopathology in COVID-19 patients. It is believed that new discoveries in the specific mechanisms linking inflammasomes with COVID-19 development could provide a more thorough understanding of SARS-CoV-2 immunopathogenesis.

Manipulation of the inflammasome signaling exerts a protective effect against COVID-19 by restricting inflammasome activation, cytokine release or SARS-CoV-2-induced pyroptotic cell death. Inflammasomes have become an attractive drug target to conquer COVID-19. Although some inflammasome-targeted molecules have potential clinical utility in treating COVID-19, their mechanisms of action, toxicity and adverse effects remain to be adequately explored. Currently, a multitude of drug candidates are still in preclinical trials, and well-controlled clinical studies are urgently warranted to evaluate their genuine therapeutic effect. Given the crucial role of the inflammasome pathway in resisting viral infection, inflammasome-directed interventions should be used with caution. The timing of intervention with inflammasome antagonists must be carefully determined. Furthermore, the upstream signaling cascades that coordinate the inflammasome machinery may also represent a therapeutic target in COVID-19 treatment. It is reported that the inflammasome priming is dictated by diverse signaling pathways, such as the autophagic flux, IL-1R, MAPK, NF-κB, TLR and TNF receptor (TNFR) (213). Specific signaling pathways (e.g., MAPK and TLR) can activate inflammasomes in response to viral infections, while negative regulatory cascades (e.g., autophagy and AMP-activated protein kinase (AMPK)) work to sustain an appropriate immune defense and protect against inflammation-inflicted organ damage by suppressing over-activation of inflammasomes. These signal transduction cascades may interact with each other to control inflammasome assembly. It is necessary to characterize the complex regulatory networks underlying inflammasome activation. The impact of SARS-CoV-2 on the activity of inflammasome-regulating pathways is an important area for further research. Importantly, autophagy and the inflammatory response have a mutually antagonistic relationship. Inducing autophagy can inhibit SARS-CoV-2-induced inflammasome formation, while blocking autophagy prevents viral immune evasion (214). The interconnection between autophagy and inflammation during COVID-19 pathogenesis deserves considerable attention. It is supposed that the combinational therapies of NLRP3 inhibitors with autophagy regulators hold great promise for strengthening antiviral responses to achieve a better therapeutic effect, which awaits further verification in the clinic. In conclusion, an in-depth investigation on the host-SARS-CoV-2 interaction will provide a theoretical basis for unearthing new treatment options in the future that could be exploited to relieve COVID-19-related complications.

## Author contributions

MW and PL conceived and supervised the project. MW wrote the manuscript and drew the figures. FY and WC searched and analyzed the literatures. YZ and LZ revised the manuscript. All authors contributed to the article and approved the submitted version.
